# First Reported Case of Imported Hantavirus Pulmonary Syndrome in Europe

**DOI:** 10.3201/eid0801.010367

**Published:** 2002-01

**Authors:** Bernadette Murgue, Yves Domart, Daniel Coudrier, Pierre E. Rollin, Jean Paul Darchis, Dominique Merrien, Hervé G. Zeller

**Affiliations:** * Institut Pasteur, Paris, France; †Centre Hospitalier, Compiègne, France;; ‡ Centers for Disease Control and Prevention, Atlanta, Georgia, USA

**To the Editor:** We report the first imported case of hantavirus pulmonary syndrome (HPS) in Europe. The patient, a 58-year-old man, traveled in Chile and Argentina from February 2 to March 2, 2001. A keen amateur botanist, he spent several days (February 17-23) on foot in Cohaique, Puerto Monte, and the surrounding rural areas collecting plant material. On February 24 and 25, he traveled by bus to Bariloche, Argentina. On March 3, shortly after he returned to France, he had a high fever (40°C to 41°C) with myalgia and headache. On March 18, he had dyspnea, which increased during the next 2 days. On March 21, he was hospitalized in the intensive care unit of the Centre Hospitalier de Compiègne, France.

On admission, the patient’s fever was still high (40°C), there was severe hypoxemia with bilateral diffuse pulmonary infiltrates, a tachyarrhythmia with auricular fibrillation and gallop, and conjunctival injection. Laboratory results indicated mild renal insufficiency (urea 12.5 mmol/L; creatininemia 180 μmol/L), hepatic cytolysis (serum glutamic-oxaloacetic transaminase 236 and serum glutamic-pyruvic transaminase 72), a moderate thrombocytopenia (platelet counts 86 000/mm^3^), an inflammatory syndrome (C-reactive protein 272 mg/L), and a capillary leak syndrome (hematocrit 49%; albuminemia 20 g/L). On the night after admission, an aggravation of the cardiac function with myocarditis developed; it responded quickly to symptomatic treatment. The patient’s condition improved steadily on the following days with a reduction of the pulmonary manifestations, and he was discharged on April 2.

Blood samples obtained on March 21 and March 29 were tested for the presence of antibodies to hantaviruses (*Puumala*, *Hantaan*, *Sin Nombre*, [SNV] *Seoul*, and Laguna Negra) by immunoglobulin (Ig) M-capture and IgG enzyme-linked immunosorbent assay. IgM antibodies were detected for all these antigens on the first sample, but there was no increase on the second sample. A substantial increase in IgG titer for SNV and Laguna Negra antigens was observed from the first to the second sample, but not for the other antigens. The virus could not be detected either by reverse transcription-polymerase chain reaction or by inoculation into cell culture (three passages). Since the identification in 1993 of SNV as the cause of HPS (*1*), numerous cases of this disease have been confirmed in various regions of North and South America. The first HPS cases associated with *Andes virus* in Argentina (*2*) were observed in 1995. Since then, more than 500 HPS cases have been reported in six countries of South America (Argentina, Bolivia, Brazil, Chile, Paraguay, and Uruguay), with mortality rates ranging from 30% to 70%.

Hantaviruses are rodent-borne, and each is associated with a specific rodent. Sigmodontine rodents are the vectors of hantaviruses associated with HPS. Infections are most frequently transmitted by inhalation of virus-contaminated aerosols of rodent excreta, but human-to-human transmission has also been described (*3*).

The patient described here was probably infected in Chile and more likely in the Puerto Monte area, where HPS cases were reported in 2001. Unfortunately, virus could not be detected because the first blood sample was obtained 2 weeks after onset of fever.
